# Neural Encoding of Acupuncture Needling Sensations: Evidence from a fMRI Study

**DOI:** 10.1155/2013/483105

**Published:** 2013-08-25

**Authors:** Xiaoling Wang, Suk-Tak Chan, Jiliang Fang, Erika E. Nixon, Jing Liu, Kenneth K. Kwong, Bruce R. Rosen, Kathleen K. S. Hui

**Affiliations:** ^1^Department of Radiology, Guang An Men Hospital, China Academy of Chinese Medical Sciences, Beijing 100053, China; ^2^Athinoula A. Martinos Center for Biomedical Imaging, Department of Radiology, Massachusetts General Hospital and Harvard Medical School, Charlestown, MA 02129, USA

## Abstract

*Deqi* response, a psychophysical response characterized by a spectrum of different needling sensations, is essential for Chinese acupuncture clinical efficacy. Previous neuroimaging research works have investigated the neural correlates of an overall *deqi* response by summating the scores of different needling sensations. However, the roles of individual sensations in brain activity and how they interact with each other remain to be clarified. In this study, we applied fMRI to investigate the neural correlates of individual components of *deqi* during acupuncture on the right LV3 (Taichong) acupoint. We selected a subset of *deqi* responses, namely, pressure, heaviness, fullness, numbness, and tingling. Using the individual components of *deqi* of different subjects as covariates in the analysis of percentage change of bold signal, pressure was found to be a striking sensation, contributing to most of negative activation of a limbic-paralimbic-neocortical network (LPNN). The similar or opposite neural activity in the heavily overlapping regions is found to be responding to different needling sensations, including bilateral LPNN, right orbitofrontal cortex, and bilateral posterior parietal cortex. These findings provide the neuroimaging evidence of how the individual needle sensations interact in the brain, showing that the modulatory effects of different needling sensations contribute to acupuncture modulations of LPNN network.

## 1. Introduction

The needling sensation of *deqi*, a psychophysical response, is considered by traditional Chinese medicine to play a key role in the clinical efficacy of acupuncture [1–4]. *Deqi* is a composite of a series of needling sensations which include but are not limited to aching, pressure, soreness, heaviness, fullness, temperature change (warmth or coolness), numbness, tingling, and dull pain [2, 3, 5]. It has been demonstrated that the *deqi* sensations during acupuncture stimulation are conveyed by different nerve fiber systems [6]. For example, A*β* fibers convey numbness. Heaviness and fullness are mediated by A*δ* fibers [6]. However, the link between the needling sensation and the acupuncture effect on the brain remains an ongoing area of research. Moreover, the different components of *deqi* may attribute to effective treatment in some disorders. It has been demonstrated that numbness and soreness but not stabbing, throbbing, tingling, burning, heaviness, fullness, or aching are correlated with clinical efficacy of analgesia [4]. In this paper we investigated how components of the *deqi* sensation were individually related to the brain responses to acupuncture.

Previous neuroimaging fMRI and PET research has been studying the brain responses to acupuncture in multiple disorders which included pain, stroke, Parkinson's disease, functional dyspepsia, and Alzheimer disease [7–13]. A few acupuncture imaging reports accounted for the needling sensation [8, 9, 14–18]. A number of fMRI studies on healthy subjects including ours have consistently revealed that acupuncture with *deqi* induced extensive negative BOLD signal change (deactivation) of a limbic-paralimbic-neocortical network (LPNN) and positive BOLD signal change of somatosensory regions of the brain [9, 14–16, 19, 20]. Both commonality and specificity were observed in brain responses to acupuncture at different acupoints [16, 17, 21]. It was reported that the sensation of sharp pain and overall *deqi* were associated with separate patterns of brain activity [9, 14–16, 22]. The previous literatures has reported so far only the relationship between brain responses and overall *deqi* sensation. However, questions on the roles of individual needling sensations of *deqi *in brain activity and how they interact with each other remain to be clarified, especially the correlation with negative or positive brain activations.

In the present study, we attempted to characterize the brain response to a subset of needle sensations relating to *deqi* during the manual acupuncture at right LV3 acupoint (Taichong) on the dorsum of distal foot, with the primary purpose of confirming the hypothesis that each individual needling sensation may correspond with a distinct map of brain responses to acupuncture. The five selected sensations are pressure, numbness, heaviness, fullness, and tingling. The other *deqi* sensations related with pain, including aching, soreness, dull pain, warmth, or coolness were investigated in another separate paper. The differences in the pattern of *deqi*, including frequency and intensity in individual sensation, were used to discriminate between acupuncture and simple tactile stimulation used as control. We hypothesized that the modulatory effects of different needling sensations contribute to acupuncture modulations of LPNN network. To our knowledge, we are the first team to explore the relationship between individual components of *deqi* and brain activity during acupuncture.

## 2. Materials and Methods

### 2.1. Subjects

In the present study, we extracted data from a larger project that investigated the brain effect of acupuncture at the Athinoula A. Martinos Center for Biomedical Imaging at Massachusetts General Hospital. This study included 37 acupuncture-naïve and right-handed healthy subjects (30 subjects for acupuncture stimulation, 19–47 years old, mean ± SD 28.6 ± 8.05, 14M/16F; 15 subjects for tactile stimulation, 21–45 years old, mean ± SD, 28 ± 7.74, 4M/11F). Eight subjects had acupuncture stimulation and tactile stimulation in the same session. Six subjects had twice acupuncture stimulations and eight subjects had performed twice tactile stimulations for different objectives, such as the comparison of real acupuncture and sham acupuncture, different acupoints, different acupuncture stimulations. The study was in compliance with the Code of Ethics of the World Medical Association (Declaration of Helsinki) and the standards established by the Institutional Review Board of the hospital and the National Center of Complementary and Alternative Medicine (NCCAM) of the NIH. Subjects were screened to exclude neurological, mental and medical disorders, drug abuse, history of head trauma with loss of consciousness, and contraindications for exposure to high magnetic field. All experimental procedures were explained to the subjects, and signed informed consent was obtained prior to participation in the study.

### 2.2. Acupuncture and Tactile Stimulations

**Figure 1:**
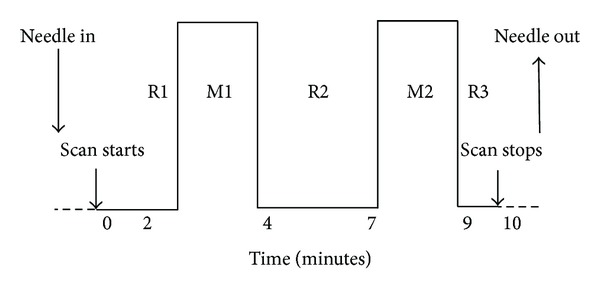
Paradigms: in each fMRI session, two periods of 2-minute acupuncture stimulation were interleaved with 3 periods of rest which lasted 2-3 minutes each. The paradigms were identical in both acupuncture and tactile stimulation.

During a single session, we administered acupuncture to LV3 on the right dorsum of distal foot using sterile, single-use, stainless steel acupuncture needles (0.20 mm diameter) (KINGLI Medical Appliance Co., Wuxi, China). Stimulation was enhanced with manipulation of the needle to elicit *deqi, *the composite of unique sensations related to efficacy according to TCM [2]. To avoid noxious pain, we tested the subject's tolerance to needle manipulation after inserting the needle at the acupoint. During the ten-minute scan, the needle was rotated approximately 180° in each direction, with even motion at the rate of 1 Hz, for two minutes during the two stimulation periods and left in place during the three rest periods (Figure 1). A licensed acupuncturist (JL) with more than 25 years of clinical acupuncture experience administered acupuncture for all subjects.

Tactile stimulation over LV3 on the right foot was used as a control for expectation and superficial sensory evaluation, as reported previously [2, 9, 15]. The skin over the acupoint was tapped gently with a 5.88 von Frey monofilament using the same paradigm as acupuncture.

### 2.3. Psychophysical Response: Needling Sensation of *Deqi*

The subjects were told that acupuncture would be performed at point using different techniques; while lying in the supine position in the scanner subjects were not able to see where the acupuncturist was working. At the completion of each scan, the subject was asked to report a full set sensations of aching, soreness, pressure, heaviness, fullness or distension, warmth or coolness, numbness, tingling, dull pain, and sharp pain and to rate each sensation, if it was experienced, on a scale of 1 to 10 [2]. If the subjects did not feel the sensation, it was noted as 0. Psychophysical data from only a subset of selected five sensations, including pressure, numbness, heaviness, fullness, and tingling, were analyzed, and the results were reported here.

### 2.4. fMRI Acquisitions

fMRI was performed on a 1.5 Tesla scanner (Siemens Sonata, Erlangen, Germany) equipped with a standard quadratic head coil. The subjects lay supine with earplugs to suppress scanner noise and cushions to immobilize the head. We acquired (1) standard high-resolution sagittal images with a T1-weighted 3D-MPRAGE sequence, and (2) whole-brain BOLD fMRI images encompassing the brain stem with a gradient-echo echo planar imaging (EPI) sequence (TR = 4000 ms, TE = 30 ms, flip angle = 90°, FOV = 200 mm, matrix = 64 × 64, thickness = 3 mm, gap = 0.6 mm), while the subject was administered acupuncture at the LV3 acupoint. Each fMRI run lasted 10 minutes.

### 2.5. Psychophysical Data Analysis

The chi-Square tests were performed for comparing the frequency of individual needling sensation between acupuncture and tactile stimulation using SPSS 19.0 (Chicago, IL, USA). Mann-Whitney *U* tests were performed for comparing the intensity of individual sensation between the acupuncture and tactile stimulation using SPSS 19.0 as well.

### 2.6. fMRI Data Analysis

All fMRI data were analyzed using the Analysis of Functional NeuroImage (AFNI) software package [23]. The first 15 volumes acquired in the first minutes of each functional dataset were discarded to eliminate the drifting of MR signals commonly seen at the beginning of acupuncture fMRI scans. Each functional dataset was motion-corrected, registered onto the subject's anatomical scan, transformed to the standardized space of Talairach and Tournoux [24], spatially smoothed with a Gaussian filter of full-width half-maximum 5 mm, and normalized to its mean intensity value across the time series. Multiple regression analysis was performed to identify brain areas showing change in the MR signal as a result of needle manipulation during acupuncture periods (ACUP), using as reference the needle left in place during the rest periods (REST). The six motion parameters were included as regressors for the removal of residual motion correlated activity.

Brain volumes with percent MR signal change to acupuncture from different subjects were then grouped and analyzed with Analysis of Covariance (ANCOVA), where the scores of 5 individual needling sensations (pressure, numbness, heaviness, fullness, and tingling) were included as covariates. The same group analysis was applied onto the brain volumes with percent MR signal change to tactile stimulation. The statistical parametric maps showing the percent MR signal change to acupuncture/tactile stimulation with respect to individual needling sensations were obtained.

In the group analysis, multicollinearity may happen when one or more of the independent sensation scores are highly correlated with one or more of the other independent sensation scores. To reliably examine the perfect or near-perfect multicollinearity, we used the variance inflation factors (VIF) by regressing scores of each sensation as a dependent variable on the scores of all the other sensations as independent variables. VIF measures the seriousness of the multicollinearity among the regressors and a VIF of 5 or above indicates a multicollinearity problem [25]. The VIF for the sensation scores in this study ranged from 1.16 to 3.894 (Table 1). Although some of the VIF were slightly higher when regressing numbness, pressure, and tingling, their values were below 5 indicating that the multicollinearity may not cause problem.

To protect against type I error, we set an individual voxel probability threshold of *P* < 0.02 to correct the overall significance level to *α* < 0.05 using Monte Carlo simulation [26]. Based on Monte Carlo simulation with 1000 iterations processed with ClusterSim program [27], the overall corrected threshold of the group activation maps for acupuncture and tactile stimulation was *P* < 0.05 with cluster volume of 108 mm^3^, and uncorrected *P* < 0.02. The group activation maps were then overlaid on the high-resolution anatomical map of the cohort in the standardized Talairach space [24]. Anatomical localization and masking of the functional data were determined by both Talairach coordinates and direct inspection.

## 3. Results

### 3.1. Psychophysical Response

**Figure 2:**
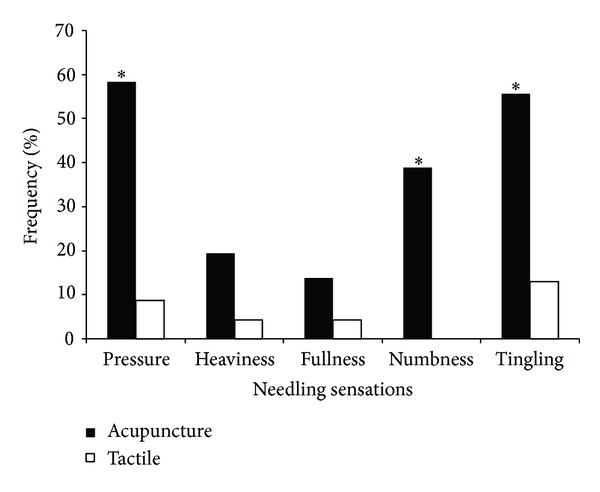
Comparison of the frequency of different sensations between acupuncture and tactile stimulation. In acupuncture, pressure was the most common sensation. The frequency of pressure, numbness, and tingling during acupuncture was more common than that during tactile stimulation. *P < 0.05.

Thirty-six psychophysical datasets during acupuncture stimulation at LV3 and twenty-three psychophysical datasets during tactile stimulation were acquired. During acupuncture, more subjects experienced pressure (58.3% versus 8.7%), tingling (55.6% versus 13.0%), and numbness (38.9% versus 0%) compared with tactile stimulation (*P* < 0.05) (Table 2, Figure 2). No significant difference in the number of subjects experiencing heaviness (19.4% versus 4.3%) and fullness (13.9% versus 4.3%) was found between acupuncture and tactile stimulation (*P* > 0.05).

**Figure 3:**
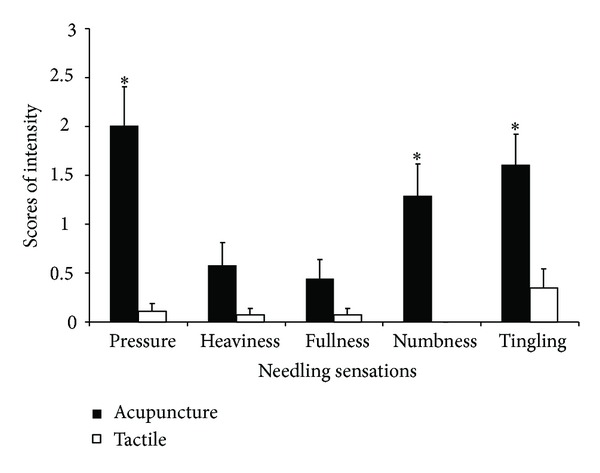
Comparison of the intensity of different sensations between acupuncture (*n* = 36) and tactile stimulation (*n* = 23). The intensity of pressure, numbness, and tingling during acupuncture was greater than that during tactile stimulation. The bar showed standard error of mean of the scores. *P < 0.05.

The intensities of individual sensations in subjects were variant. The scores were not in normal distribution in each group. The Mann-Whitney *U* tests were used to compare the difference between two groups. The intensity of pressure (2.01 ± 0.40 versus 0.11 ± 0.07), numbness (1.29 ± 0.33 versus 0), and tingling (1.61 ± 0.31 versus 0.35 ± 0.19) was found to be greater for acupuncture relative to tactile stimulation (*P* < 0.05) (Table 3, Figure 3). No significant difference in the intensity of heaviness (0.58 ± 0.23 versus 0.07 ± 0.06) and fullness (0.44 ± 0.2 versus 0.07 ± 0.06) was found between acupuncture stimulation and tactile stimulation (*P* > 0.05).

### 3.2. fMRI Data: Brain Response

Thirty-six fMRI datasets during acupuncture stimulation at LV3 and twenty-three fMRI datasets during tactile stimulation at LV3 were acquired. Psychophysical responses acquired immediately after the fMRI sessions were included as covariates in this analysis.

#### 3.2.1. Mean Effect of Overall Brain Activity during Acupuncture and Tactile Stimulation

**Figure 4:**
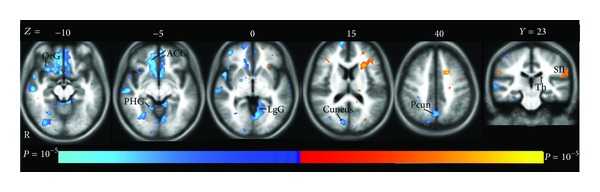
The mean brain positive (yellow) and negative (blue) bold responses to acupuncture stimulation. The extensive deactivation showed limbic-paralimbic-neocortical network (LPNN), such as anterior cingulated cortex (ACC), parahippocampal gyrus (PHG), lingual gyrus (LgG), precuneus (Pcun), and cuneus. The orbital gyrus (OrG) also showed deactivation. Left thalamus (Th) and secondary somatosensory cortex (SII) demonstrated activation.

Consistent with our previous studies [9, 17], acupuncture stimulation at LV3 elicited extensive deactivation in LPNN, such as anterior cingulate cortex (ACC), medial temporal lobe (temporal pole, amygdala, hippocampus, and parahippocampus gyrus), and precuneus (Figure 4). Most of the deactivations showed bilateral distribution. Sparse positive activations were identified in left splenium of corpus callosum, left thalamus, left anterior, and bilateral superior segments of circular sulcus of the insula, left postcentral sulcus, right superior frontal gyrus, bilateral supramarginal gyrus, and bilateral cerebellar cortex. The summary of the regions showing positive and negative activations elicited by acupuncture stimulation is shown in Table 4.

Tactile control stimulation elicited deactivation in the aforementioned areas far less than acupuncture stimulation. Positive activations were also found in the left inferior segment of circular sulcus of the insula, left postcentral sulcus, and left supramarginal gyrus, but the extent was much smaller than that in acupuncture stimulation. The summary of the regions showing positive and negative activations elicited by tactile control stimulation is shown in Table 5.

#### 3.2.2. Brain Activity Associated with Individual Sensation Related to *Deqi* during Acupuncture Stimulation

**Figure 5:**
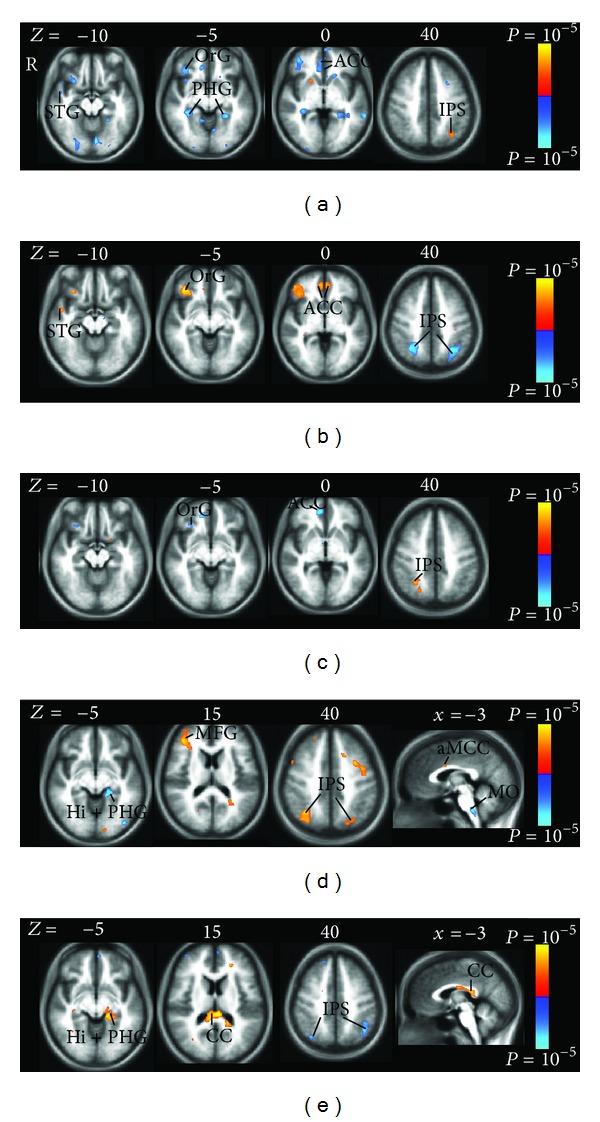
Brain positive (yellow) and negative (blue) bold responses associated with the intensity of individual needling sensation during acupuncture stimulation. The individual sensations are pressure (a), heaviness (b), fullness (c), numbness (d), and tingling (e). The brain regions associated with differential individual sensation are partly overlapped, such as bilateral ACC, right lateral prefrontal cortex (OrG, orbital gyrus), bilateral medial temoral cortex (Hi, hippocampus; PHG, parahippocampal gyrus), and bilateral posterior parietal cortex (IPS, intraparietal sulcus). (a) Pressure contributed to the negative activity in the LPNN network and showed symmetric distributions, such as ACC and PHG. (b) Heaviness showed positive activity in the bilateral ACC, right superior temporal gyrus (STG), and right OrG and negative activity in the bilateral IPS. Heaviness and pressure showed anticorrelated impact on the regions mentioned previously. (c) Fullness was associated with the negative activity in the right ACC and OrG and the positive activity in the right IPS. (d) Numbness showed positive activity in the right middle frontal gyrus (MFG), right anterior middle cingulate cortex (aMCC), and bilateral IPS and negative activity in the left hippocampus (Hi), left PHG, and medulla oblongata (MO). (e) Tingling showed positive activity in the posterior corpus callosum (CC) but negative activity in the posterior parietal cortex (IPS). Tingling and numbness showed anticorrelated impact on bilateral IPS, left Hi, and left PHG.

Comparing with tactile stimulation, more needlingsensations during acupuncture showed extensive significant association with certain brain regions. In this paper, we focused on the brain responses which were correlated with the five sensations including pressure, fullness, heaviness, numbness, and tingling reported by the subjects (Table 4, Figure 5). The brain regions associated with differential individual sensation are partly overlapped, such as bilateral ACC, right lateral prefrontal cortex, bilateral medial temporal cortex, and bilateral posterior parietal cortex.

**Figure 6:**
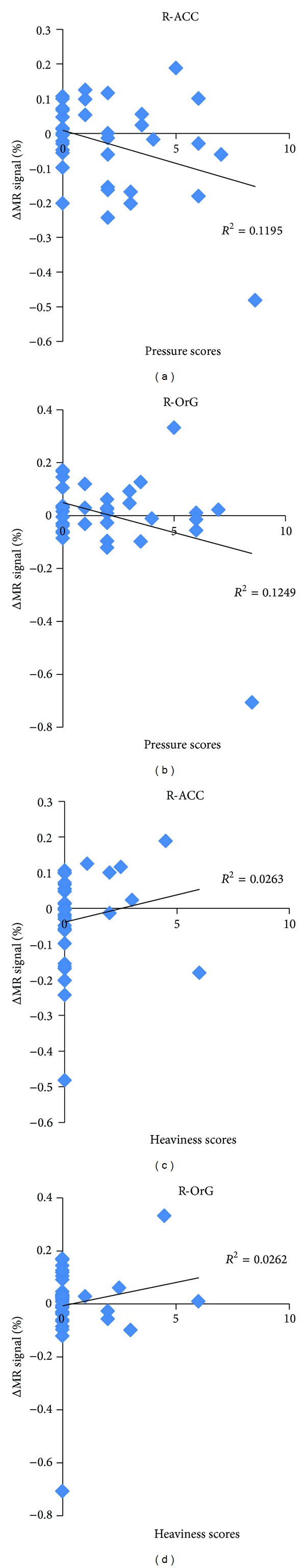
Variance in activity is accounted for by scores of intensity of pressure and heaviness during acupuncture. The negative correlation between the score of pressure and mean MR signal percentage change was shown in the (a) right ACC (anterior cingulate cortex) and (b) right OrG (orbital gyrus). The positive correlation between the score of heaviness and mean MR signal percentage change was shown in the (c) right ACC and (d) right OrG.

The pressure elicited negative activation bilaterally in LPNN network, such as ACC and medial temporal cortex (hippocampus and parahippocampus). Reduced brain activity was also observed unilaterally in left superior frontal gyrus, left straight gyrus, right orbital gyrus and sulcus, right superior temporal gyrus and sulcus, right temporal pole, and right anterior segments of circular sulcus of the insula (Table 4, Figures 5(a), 6(a), 6(b)). Increased brain response was sparsely shown in the left intraparietal sulcus (IPS), left transverse parietal sulci, and right superior segments of circular sulcus of the insula.

While fullness also contributes to the negative activations at the right lateral prefrontal cortex and ACC as in pressure sensation, heaviness demonstrates positive activity at the same areas (Table 4, Figures 5(b), 5(c)). A number of brain regions showing negative activations with the increased intensity in pressure sensation were found to have positive activations with the increased intensity in heaviness sensation (Figures 6(c), 6(d)). These regions include the bilateral ACC, right inferior frontal cortex (orbital gyri and sulcus), left superior frontal gyrus, right anterior segment of circular sulcus of the insula, right superior temporal sulcus, right middle temporal gyrus, and right hippocampus.

On the contrary, the negative activity related to heaviness at the posterior parietal cortex (bilateral IPS and transverse parietal sulci) overlaps with the positive activity related to numbness. Numbness decreased brain activity in the bilateral hippocampus, left parahippocampus, and left thalamus and increased brain activity in the right superior frontal gyrus and bilateral posterior parietal cortex (angular gyrus, superior parietal lobule, supramarginal gyrus, IPS, and transverse parietal sulci) (Table 4, Figure 5(d)).

Tingling sensation was correlated with the brain response mainly in two areas: positive correlation at posterior corpus callosum (posterior midbody, isthmus, and splenium) but negative correlation at posterior parietal cortex (bilateral angular gyrus, bilateral IPS and transverse parietal sulci, right superior parietal lobule, right postcentral gyrus, and left supramarginal gyrus) (Table 4, Figure 5(e)).

#### 3.2.3. Brain Activity Associated with Individual Sensation Related to *Deqi* during Tactile Stimulation

For tactile stimulation, the sensation of pressure, tingling, fullness, and heaviness had the sparse impact on the brain activity. The pressure and tingling mainly correlated with positive brain activity, while fullness and heaviness mainly demonstrated negative activity.

**Figure 7:**
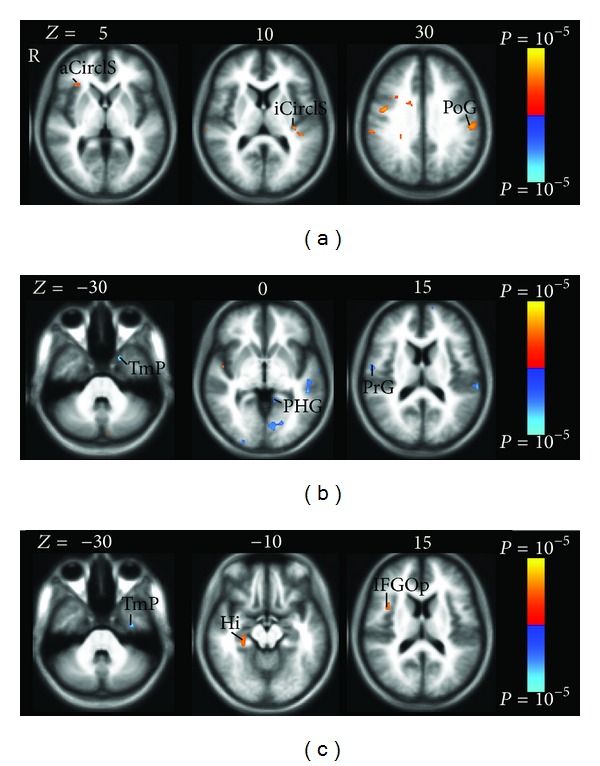
Brain positive (yellow) and negative (blue) bold responses associated with the intensity of individual sensation during tactile stimulation. The individual sensations are pressure (a), heaviness/fullness (b), and tingling (c). (a) Pressure associated with positive activation in the sensorimotor regions, such as right anterior segment of the circular sulcus of the insula (aCirclS), left inferior segment of the circular sulcus of the insula (iCirclS), and left postcentral gyrus (PoG). (b) Heaviness and fullness had the same impact on the negative activation in the right precentral gyrus (PrG), left parahippocampal gyrus (PHG), and left temporal pole (TmP). (c) Tingling was associated with sparse positive brain activity in right opercular part of the inferior frontal gyrus (IFGOp) and right hippocampus (Hi) and negative brain activity in left Tmp.

Pressure significantly is associated with positive activation in the sensorimotor regions, such as bilateral precentral gyrus, bilateral postcentral gyrus and sulcus, right anterior and superior segments of circular sulcus of the insula, and left inferior segment of circular sulcus of the insula (Table 5, Figure 7(a)).

Heaviness and fullness had the same impact on the brain activity because only one subject had the two sensations with the same intensity score. The two sensations were mainly associated with the negative activity in the bilateral superior temporal gyrus, right precentral gyrus, right central sulcus, left operculum part of the inferior frontal gyrus, left temporal pole, left parahippocampus, and left lingual gyrus (Table 5, Figure 7(b)).

Tingling was mainly associated with sparse positive brain activity in right anterior and superior segments of circular sulcus of the insula, right opercular part of the inferior frontal gyrus, and right hippocampus (Table 5, Figure 7(c)).

## 4. Discussion

*Deqi* response [1–4], a psychophysical response characterized by a spectrum of different needling sensations, is essential for Chinese acupuncture clinical efficacy. A number of fMRI studies including ours have revealed brain activity to acupuncture stimulation [9, 14–16, 19, 20]. However, it has not been reported the impact of these individual sensations of *deqi* on the brain activity during acupuncture stimulation. In this part of the study, we applied fMRI to investigate the neural correlates of five individual sensations including pressure, heaviness, numbness, fullness, and tingling, of *deqi* response during acupuncture at LV3 acupoint. The major findings in the present study included that (1) the pressure sensation was associated with the extensive deactivation of LPNN network during acupuncture; (2) partial overlapping of the positive or negative activity at some of the brain regions associated with the five individual sensations. They included bilateral LPNN, the right lateral orbitofrontal cortex and bilateral posterior parietal cortex. Some needling sensations showed anticorrelated association within the same brain regions; (3) the tingling sensation showed positive correlation with the brain activity in the bilateral posterior corpus callosum. These findings provide the neuroimaging evidence showing how the different individual needle sensations of *deqi* could both interact differently with the brain and share common interaction with the brain.

### 4.1. The Pressure Sensation on the Brain Activity of LPNN and Default Mode Network

According to the psychophysical responses, pressure stood out as the most important needling sensation of acupuncture in this study. In addition to the highest frequency (Figure 2) and intensity (Figure 3) among all the related sensations of *deqi* response, pressure also contributed significantly to the extensive deactivation of LPNN, by which acupuncture may mediate its antipain, antianxiety, and other diverse modulatory effect [16].

The salient brain regions that correlate with pressure included bilateral ACC, right inferior frontal cortex, bilateral hippocampus and parahippocampus, bilateral lingual gyrus, right temporal pole, and right insula (Table 4, Figure 5(a)). Many of these brain regions associated with pressure have been shown to be overlapped with those in the default mode network [9, 18, 28]; the integrity of default mode network has been postulated to be central to the balance of global neurological function and the maintenance of health [29].

On the contrary, the increase in the intensity of pressure sensation during tactile stimulation increased brain activity in the sensorimotor regions, such as precentral cortex, postcentral cortex, and insula (Table 5, Figure 7). Such an extreme difference is likely due to the stimulation on the nerves at the cutaneous level during tactile stimulation compared to deep nerve stimulation during manual acupuncture. The LV3 acupoint is located on the dorsum of the foot in the fossa distal to the junction of the first and second metatarsal bones 2 *cuns *(the proportional unit of accurate location of the acupuncture points) above the web of the toe. In tactile stimulation, the mechanoreceptors in the superficial layers transmit pressure sensation to sensorimotor regions by A*δ* and A*β* fibers [30]. In acupuncture stimulation, the needle passage includes skin, subcutaneous tissue, the lateral side of the extensor hallucis brevis muscle, deep peroneal nerve, first dorsal metatarsal artery and vein, and first dorsal interosseous muscle [31]. With the deep stimulation at LV3, gentle and repetitive manipulation producing mechanical pressure and tissue distortions activates more mechanoreceptors and nociceptors that are innervated by thin myelinated A*δ* and C fibers [32]. Both frequency and intensity of pressure in the acupuncture were therefore higher than those in the tactile stimulation, which was also shown in our analysis of psychophysical data (Figures 2, 3). This is also supported by an earlier human acupuncture study at LI4 (Hegu) on hand, which has the similar tissue composition as LV3 on foot. Chiang et al. found that acupuncture analgesia was completely abolished by blockade of deep nerve branches innervating muscle fibers but not cutaneous nerve fibers [33]. Moreover, the studies on pressure sensation elicited by nonacupuncture mechanical stimulation in deep and superficial tissue had the similar findings [34, 35]. Graven-Nielsen et al. [34] found that the nonpainful pressure sensation can be evoked mechanically from human muscle tissue with complete cutaneous anesthesia. They concluded that the nonpainful pressure sensation is mediated by A*δ* and C afferents involving low-threshold mechanoreceptors in the deep tissues. The pressure sensation induced by the temporal summation of mechanical stimulation in deep tissue was shown to be more potent than that in the pure skin stimulation, suggesting that A*δ* and C muscle afferent fibers mediate the deep tissue pressure sensation [35].

In addition to the A*δ* and A*β* fibers located in superficial layers, our findings indicated that pressure sensation elicited by acupuncture stimulation mainly involved A*δ* and C afferent fibers in deep tissues. The studies using EEG [36] and MEG [37, 38] showed that selective stimulation of C-fibers induced the ultra-late evoked brain potentials at the ACC [36, 38], posterior parietal cortex [37], insula, and somatosensory cortex [37, 38]. An recent fMRI study showed that increased activity in the right frontal operculum, inferior frontal cortex and anterior insula to C-fiber alone stimulation as compared to A*δ*-fiber alone stimulation. The simulation of A*δ*-fiber or C-fiber were both associated with activation in ACC, SMA and thalamus [39]. These brain regions associated with C-fiber/A*δ*-fiber in the experimental studies are consistent with our findings in a majority, such as ACC, inferior frontal cortex, and insula. Moreover, a co-stimulation of C- and A-fiber input as produced by usual large-area laser stimulations prevents the recording of ultralate evoked brain potentials (ULEPs), potentials that can be recorded in response to selective stimulation of C-fibers [36]. The negative activity associated with pressure sensation may be the results of the costimulation of C- and A*δ*-fiber input, leading to a repression of the central processing of the C-fiber input [39].

### 4.2. Interplay of the Sensations Pressure, Heaviness, Fullness, Tingling, and Numbness to the Overlapped Brain Regions

The richness of sensory experience is obviously conveyed not by a single receptor or sensory axon but by populations of nerve fibers [30]. It is well accepted that a wide spectrum of myelinated and unmyelinated nerve fibers in cutaneous and/or muscular layers are involved during acupuncture stimulation [6, 40–43]. In a human acupuncture study by means of analyzing power spectrum of the unit discharges with FFT, Wang and colleagues found the relationship between heaviness and fullness and A*δ* nerve fibers [6]. In our study, we found that pressure, heaviness, and fullness were associated with heavily overlapping neural activity in ACC, inferior frontal cortex, and insula (Table 4, Figures 5(a), 5(b)). It is consistent with brain response to the stimulation of A*δ* and C fiber discussed previously. Compared with pressure, heaviness demonstrated anticorrelated positive activity, while fullness showed similar negative activity in these brain regions. It is possible that fullness also is involved in C-fiber.

All the five sensations demonstrated the associations with brain activity in the posterior parietal cortex including the superior parietal lobule, the inferior parietal lobule, and IPS (Table 4, Figure 5). The posterior parietal cortex receives somatosensory and/or visual input. IPS is crucial for integrating these sensory information related to the body, which through motor signals controls movement of limb and eye movement [44]. The study using MEG showed that both selective stimulation of A*δ*-fiber or C-fibers induced the ultra-late evoked brain potentials at posterior parietal cortex [37], which supported our findings in the sensations of pressure, heaviness, and fullness involving A*δ*-fiber and/or C-fibers. On the other hand, the relationship between the sensations of numbness and tingling and posterior parietal cortex also can be supported by tingling and numbness of limbs commonly found in patients with parietal lobe epilepsy [45, 46].

A number of studies have shown that acupuncture elicits clinical effects via the activation of afferent nerve fibers innervating the skin and muscles [41–43, 47, 48]. The somatic afferent information of nerve fibers has various effects on body function, including analgesia, somatic, autonomic and hormonal response [41, 42, 47, 48]. For example, in a human study on the characteristics of afferent fiber innervation on ST36 (zusanli), which has a significant suppressive effect on jaw movement response (JMR) and electromyogram of digastric muscle induced by acupuncture stimulation, Lu found that these effects were weakened or abolished by sectioning the peroneal nerve and blocking A*β* and some A*δ*-fiber. They had a conclusion that the predominance of large afferent fibers was thought to be one of the fundamental characteristics of the acupoint [43].

In line with these studies, these brain regions associated with needling sensation play a role in a wide variety of emotional regulation, cognition, memory, and pain modulation [49–54].The present results in the humans clearly show how these nerve fibers impact on the central nervous system.

### 4.3. White Matter (Corpus Callosum) Activity during Acupuncture Stimulation

We found that tingling sensation demonstrated significant positive correlation at posterior corpus callosum (posterior midbody, isthmus, and splenium) (Figure 5(e)), which is the principal white matter fiber bundle connecting neocortical areas of the two hemispheres. As the white matter, the corpus callosum is seldom reported in the acupuncture fMRI studies, though a growing number of studies are reporting the fMRI activation in white matter, specifically corpus callosum [55–59]. Our findings can be supported by that the lesions of splenial corpus callosum are responsible for numbness and tingling sensations of unilateral limb or face in patients [60, 61], where the corpus callosum is structurally connected to the functional network of gray matter regions that are involved in the interhemispheric transfer task [57]. Human and monkey studies have shown that the posterior corpus callosum contains connections between the parietal and occipital cortices and plays a role in transferring sensory information [62–66]. Consistent with these results, all the five needling sensations in the present study were associated with posterior parietal cortex as mentioned previously, especially tingling and numbness, supporting that the activation in corpus callosum is the important hemodynamic response of acupuncture stimulation instead of artifact. We further postulate that the corpus callosum may be an important component of acupuncture convey pathway.

### 4.4. Differences in Brain Activity: Mean Effect versus Individual Needling Sensations Effect of Acupuncture Stimulation

The overlapped regions of the effect of individual needling sensations of acupuncture stimulation provide the details in the modulatory effect of the mean effect. On the other hand, some important brain regions, which are salient in the individual needling sensations effect, might show small or no significant activity in the mean effect (Figures 4, 5). For example, the posterior corpus callosum demonstrated bilateral extensive activation associated with tingling, while the unilateral small activation shown in the mean effect may be missed as a small artifact [59]. Similarly, for the region of IPS, being crucial for integrating the sensory information related to the body [44], it was extensively associated with all the five individual needling sensations. However, no significant activity was shown in the mean effect. That is why it is insufficient to study the mean effect alone for acupuncture effects.

## 5. Limitations

In the present study, we extracted data from a larger project that investigated the brain effect of acupuncture. Some of the subjects had more than once acupuncture experiments for different objectives, such as the comparison of real acupuncture and sham acupuncture, different acupoints, and different acupuncture stimulations. However, the data are qualified for the purpose of this study to evaluate the correlation between behavior response and brain response during acupuncture. Considering of the variability of needling sensation during acupuncture stimulation, the further investigation on larger sample size is warranted.

## 6. Conclusions

The similar or opposite neural activity in the heavily overlapping regions of LPNN and DMN are found responding to different sensations of *deqi* elicited by acupuncture stimulation. The posterior corpus callosum is involved in acupuncture sensation convey pathway. Our data provide the neuroimaging evidence of how the individual needle sensations of *deqi* interact in the brain during acupuncture, and the messages of individual sensation are integrated as the signals converge on processing centers in the central nervous system. It is confirmed that the different psychophysical responses are correlated with the distinct hemodynamic activities.

## Figures and Tables

**Table 1:** The variance inflation factors (VIF) by regressing scores of each sensation as a dependent variable on the scores of all the other sensations as independent variables.

	Independent variables				
	Heaviness	Fullness	Numbness	Pressure	Tingling
Dependent variable					
Heaviness	—	1.376	1.463	1.109	1.187
Fullness	1.867	—	1.641	1.424	1.164
Numbness	3.186	2.634	—	1.442	1.162
Pressure	2.823	2.671	1.686	—	1.193
Tingling	3.894	2.813	1.75	1.537	—

**Table 2:** The chi-Square tests were performed for comparing the frequency of individual sensation between acupuncture and tactile stimulation.

Deqi	*χ* ^2^	*P*
Pressure	14.537	<0.001
Heaviness	2.729	0.099
Fullness	1.398	0.237
Numbness	11.727	0.001
Tingling	9.475	0.002

**Table 3:** Mann-Whitney *U* tests were performed for comparing the intensity of individual sensation between the acupuncture and tactile stimulation.

Deqi	*Z*	*P*
Pressure	−3.93	<0.001
Heaviness	−1.698	0.09
Fullness	−1.245	0.213
Numbness	−3.358	0.001
Tingling	−3.079	0.002

**Table 4:** Summary of anatomic foci showing brain activity of acupuncture stimulation and positive and negative bold responses associated with the intensity of individual needling sensation (*P* < 0.05 corrected).

Atlas structure at the center of maximum difference		Mean fMRI response to acupuncture				Pressure				Heaviness			
		*x*	*y*	*z*	t*	*x*	*y*	*z*	t*	*x*	*y*	*z*	t*
Straight gyrus	L	−2	29	−10	−4.40	−2	59	−1	−3.64				
	R	5	11	−10	−4.65								
Superior frontal gyrus	L					−5	50	27	−3.19	−5	−14	48	2.94
	R	8	32	48	5.01								
	R	8	53	24	−4.19								
Orbital gyri	R	11	44	−13	−4.97	35	29	−7	−3.78	35	29	−7	4.52
Orbital sulci	R	17	41	−10	−3.71	32	29	−7	−3.45	38	32	−4	4.33
Anterior cingulate cortex	L	−8	32	−4	−3.17	−5	32	3	−3.08	−5	41	3	3.70
	R	8	32	−7	−5.01	8	38	3	−3.29	8	44	3	3.84
Subcallosal gyrus	L	−2	11	−7	−3.94	−2	14	3	−2.77				
	R	5	11	−10	−4.65								
Middle-anterior cingulate cortex	R												
Postcentral gyrus	R	44	−20	51	−3.40								
Postcentral sulcus	L	−47	−32	36	3.85								
Superior parietal lobule	L	−11	−65	51	−3.28								
	R	20	−74	39	−3.26								
Intraparietal sulcus	L					−35	−59	39	2.90	−38	−56	39	−4.08
	R									29	−53	42	−4.07
Angular gyrus	L					−53	−59	30	−3.44				
	R	44	−71	30	−3.25	44	−65	33	−2.72				
Supramarginal gyrus	L	−62	−32	33	4.14								
	R	56	−23	24	3.10								
Precuneus	L	−8	−62	48	−3.69					−5	−59	33	2.81
	R	5	−62	42	−3.34								
Ant. insula	L	−26	26	6	2.85								
	R	29	17	−10	−3.73	29	17	−10	−3.34	29	23	−7	3.57
Sup. insula	L	−26	20	15	4.16								
	R	35	26	15	2.82	29	23	12	2.95				
Superior temporal gyrus	R	53	8	−13	−4.92	47	17	−10	−3.23				
Superior temporal sulcus	R	53	−17	−10	−4.94	56	−2	−16	−3.80	50	−2	−10	3.41
Middle temporal gyrus	L	−62	−26	−7	−3.04	−62	−41	3	−4.16				
	R	56	2	−16	−5.19	59	−2	−19	−5.25	62	−17	−13	3.48
Hippocampus	L	−29	−23	−7	−2.72	−23	−41	−1	−3.50				
	R	23	−11	−16	−3.37	32	−35	−4	−4.45	32	−35	−4	2.87
Amygdala	R	26	−8	−16	−3.27								
Parahippocampal gyrus	L	−11	−38	−7	−4.09	−23	5	−19	−4.86				
	R	23	2	−25	−3.98	20	5	−22	−3.46				
Lingual gyrus	L	−8	−62	−1	−4.21	−5	−80	−13	−3.95				
	R	14	−47	−7	−3.65	11	−50	−4	−3.32				
Temporal pole	R	32	17	−28	−5.17	23	5	−28	−3.74				
Cuneus	L	−5	−83	12	−3.64								
	R	5	−86	12	−2.79								
Thalamus proper	L	−17	−20	18	3.09								
	R												
Corpus callosum	L	−12	−42	19	3.09								
	R												
Cerebellum cortex	L	−35	−68	−25	4.48					−8	−53	−31	2.72
	L	−11	−38	−10	−3.93	−38	−56	−25	−3.01				
	R	29	−44	−43	3.83								
	R	5	−41	−10	−3.48	41	−68	−34	−4.36				
Brain stem		5	−26	−34	−3.00								

Atlas structure at the center of maximum difference		Fullness				Numbness				Tingling			
		*x*	*y*	*z*	t*	*x*	*y*	*z*	t*	*x*	*y*	*z*	t*

Straight gyrus	L									−2	53	−7	−3.54
	R												
Superior frontal gyrus	L	−14	56	27	−2.88					−20	8	60	−3.73
	R					8	−2	57	3.69				
	R									17	44	39	−2.93
Orbital gyri	R	29	26	−10	−3.48					38	50	−1	−3.83
Orbital sulci	R	32	29	−7	−2.99								
Anterior cingulate cortex	L												
	R	11	41	−1	−4.20								
Subcallosal gyrus	L												
	R												
Middle-anterior cingulate cortex	R					5	8	30	3.61				
Postcentral gyrus	R									47	−29	54	−3.46
Postcentral sulcus	L					−56	−23	30	3.10				
Superior parietal lobule	L					−23	−71	36	2.98				
	R	20	−68	42	2.61	20	−68	48	2.96	20	−68	45	−3.38
Intraparietal sulcus	L					−20	−68	39	3.19	−44	−47	39	−3.40
	R	29	−50	39	3.71	32	−56	39	4.47	32	−65	39	−2.79
Angular gyrus	L					−29	−68	36	2.65	−44	−56	42	−3.66
	R	56	−44	27	−2.95	35	−65	39	3.55	38	−65	42	−3.37
Supramarginal gyrus	L					−62	−29	36	3.67	−44	−47	42	−2.96
	R	50	−23	18	3.31	56	−23	30	3.00				
Precuneus	L									−14	−47	57	−2.69
	R												
Ant. insula	L												
	R	29	23	−7	−2.91								
Sup. insula	L												
	R					35	26	15	3.03				
Superior temporal gyrus	R												
Superior temporal sulcus	R	47	−2	−19	−2.62	53	−14	−10	−2.85	44	−71	24	−2.59
Middle temporal gyrus	L									−62	−32	−4	−2.79
	R	62	−17	−16	−3.16	56	2	−16	−4.02				
Hippocampus	L					−20	−32	−7	−3.92	−17	−29	−7	4.69
	R					35	−32	−7	−2.56	35	−26	−7	2.90
Amygdala	R												
Parahippocampal gyrus	L					−14	−38	−7	−3.65	−14	−32	−7	3.87
	R												
Lingual gyrus	L					−11	−86	−7	2.50				
	R												
Temporal pole	R												
Cuneus	L					−5	−86	12	2.79				
	R												
Thalamus proper	L					−20	−32	−4	−3.90	−8	−32	6	5.48
	R									11	−29	15	3.56
Corpus callosum	L									−17	−35	24	5.17
	R									8	−35	18	4.70
Cerebellum cortex	L	−2	−44	−19	2.50	−47	−56	−28	3.55	−38	−71	−31	3.89
	L					−14	−38	−13	−2.75				
	R	2	−44	−19	3.12					44	−41	−40	4.00
	R					35	−56	−40	3.14				
Brain stem						8	−29	−34	4.02	−14	−23	−7	3.76

t* is the value taken from the voxel with maximal signal change.

“−” means negative bold responses.

Ant. insula: anterior segment of the circular sulcus of the insula.

Sup. insula: superior segment of the circular sulcus of the insula.

**Table 5:** Summary of anatomic foci showing brain activity of tactile stimulation and positive and negative bold responses associated with the intensity of individual needling sensation (*P* < 0.05 corrected).

Atlas structure at the center of maximum difference		Mean fmri response to tactile				Pressure				Heaviness				Fullness				Tingling			
		*x*	*y*	*z*	t*	*x*	*y*	*z*	t*	*x*	*y*	*z*	t*	*x*	*y*	*z*	t*	*x*	*y*	*z*	t*
Inferior frontal gyrus	L									−53	14	24	−4.01	−53	14	24	−4.01				
	R					56	−2	12	3.37									41	8	12	3.02
Superior frontal gyrus	L									−11	62	21	−2.91	−11	62	21	−2.91				
	R	20	−2	63	−3.20																
Precentral gyrus	L	−20	−26	63	−2.86	−56	5	24	4.64												
	R	35	−26	48	−2.78	56	−5	24	3.44	35	−26	51	−3.05	35	−26	51	−3.05				
Orbital gyri	R	14	47	−10	−3.29																
Central sulcus	L	−38	−29	57	−3.20																
	R	38	−20	42	−4.55	56	−5	24	3.44	32	−26	51	−3.02	32	−26	51	−3.02				
Postcentral gyrus	L	−38	−29	57	−3.20	−59	−17	30	3.46												
	R	47	−17	45	−3.01	50	−17	48	3.63												
Postcentral sulcus	L	−56	−23	30	3.31	−56	−20	30	4.03												
	R	26	−38	57	−3.30	53	−20	36	3.00												
Supramarginal gyrus	L	−62	−26	24	3.71	−56	−23	30	3.72												
	R					56	−29	27	3.36												
Inf. insula	L	−32	−23	9	3.20	−35	−23	9	3.79												
Ant. insula	R	29	23	6	−3.71	32	26	6	3.08									29	23	6	3.08
Sup. insula	R	35	−14	21	−3.53	35	8	15	3.02									38	11	15	3.55
Superior temporal gyrus	L	−47	8	−19	−3.62	−50	8	−4	3.55	−62	−26	12	−3.19	−62	−26	12	−3.19				
	R	59	−8	−1	−5.02	65	−32	15	3.18	62	−8	6	−3.33	62	−8	6	−3.33				
Superior temporal sulcus	L	−47	5	−19	−3.99					−47	−35	−1	−4.53	−47	−35	−1	−4.53				
	R	59	−11	−1	−4.84																
Middle temporal gyrus	L	−47	8	−22	−3.52																
	R	44	2	−25	−3.68																
Parahippocampal gyrus	L									−20	−29	−16	3.01	−20	−29	−16	3.01	−20	−14	−25	5.48
	L	−32	−17	−22	−2.95					−14	−41	−4	−3.00	−14	−41	−4	−3.00	−32	−11	−31	−4.28
Hippocampus	R	32	−26	−13	−5.38													29	−26	−13	3.86
Lingual gyrus	L	−2	−83	−7	−3.02					−8	−74	6	−3.96	−8	−74	6	−3.96				
Temporal pole	L	−47	5	−28	−3.07					−20	8	−31	−8.18	−20	8	−31	−8.18	−32	−8	−31	−5.31
	R	47	11	−25	−3.06	41	−2	−34	2.77												
Cerebellum cortex	L									−8	−77	−31	3.38	−8	−77	−31	3.38				
	L	−2	−53	−31	−3.08					−32	−47	−52	−3.95	−32	−47	−52	−3.95				
	R	2	−50	−31	−3.26	35	−53	−52	4.07												
Brain stem						−5	−29	−22	3.28												

t* is the value taken from the voxel with maximal signal change.

“−” means negative bold responses.

Inf. insula: inferior segment of the circular sulcus of the insula.

Ant. insula: anterior segment of the circular sulcus of the insula.

Sup. insula: superior segment of the circular sulcus of the insula.
